# An Axis Involving SNAI1, microRNA-128 and SP1 Modulates Glioma Progression

**DOI:** 10.1371/journal.pone.0098651

**Published:** 2014-06-24

**Authors:** Qingsheng Dong, Ning Cai, Tao Tao, Rui Zhang, Wei Yan, Rui Li, Junxia Zhang, Hui Luo, Yan Shi, Wenkang Luan, Yaxuan Zhang, Yongping You, Yingyi Wang, Ning Liu

**Affiliations:** 1 Department of Neurosurgery, The First Affiliated Hospital of Nanjing Medical University, Nanjing, China; 2 Department of Urology, Affiliated Zhongda Hospital, Southeast University, Nanjing, China; H. Lee Moffitt Cancer Center & Research Institute, United States of America

## Abstract

**Background:**

Glioblastoma is an extraordinarily aggressive disease that requires more effective therapeutic options. Snail family zinc finger 1, dysregulated in many neoplasms, has been reported to be involved in gliomas. However, the biological mechanisms underlying SNAI1 function in gliomas need further investigation.

**Methods:**

Quantitative real-time PCR was used to measure microRNA-128 (miR-128) expression level and western blot was performed to detect protein expression in U87 and U251 cells and human brain tissues. Cell cycle, CCK-8, transwell and wound-healing assays were performed. Dual-luciferase reporter assay was used for identifying the mechanism of SNAI1 and miR-128b regulation. The mechanism of miR-128 targeting SP1 was also tested by luciferase reporter assay. Immunohistochemistry and in situ hybridisation staining were used for quantifying SNAI1, SP1 and miR-128 expression levels in human glioma samples.

**Results:**

The Chinese Glioma Genome Atlas (CGGA) data revealed that SNAI1 was up-regulated in glioma and we confirmed the findings in normal and glioma tissues. SNAI1 depletion by shRNA retarded the cell cycle and suppressed proliferation and invasion in glioma cell lines. The CGGA data showed that the Pearson correlation index between SNAI1 and miR-128 was negatively correlated. SNAI1 suppressed miR-128b expression by binding to the miR-128b specific promoter motif, and miR-128 targeted SP1 via binding to the 3′-untranslated region of SP1. Moreover, introduction of miR-128 anti-sense oligonucleotide alleviated the cell cycle retardation, proliferation and invasion inhibition induced by SNAI1 shRNA. Immunohistochemistry and in situ hybridisation analysis of SNAI1, SP1 and miR-128 unraveled their expression levels and correlations in glioma samples.

**Conclusions:**

We propose that the SNAI1/miR-128/SP1 axis, which plays a vital role in glioma progression, may come to be a clinically relevant therapeutic target.

## Introduction

Gliomas, which account for most intracranial malignant tumors with high morbidity, relapse rate and mortality, can rarely be treated because of their malignancy and resistance to chemo and radiotherapy [1], [2]. In addition, gliomas cannot be completely resected because of intracranial invasion by these tumors [3], [4]. Some predictive markers, such as methylguanine-DNA methyltransferase gene promoter hypermethylation, 1p/19q loss and IDH1 mutation, assist in clinical treatment [5]–[7]. However, such treatment is insufficient. Gliomagenesis results from abnormalities in several signalling pathways, including deregulation of tumor suppressor genes and oncogenes, and other unknown pathways.

SNAI1 is a vital member of the zinc finger superfamily of proteins, which consists of SNAI1 (SNAIL), SNAI2 (SLUG) and SNAI3 (SMUC), all of which function as transcription repressors. These proteins share the evolutionarily conserved N-terminal SNAG domain (mediating transcription repression) and C-terminus zinc fingers (binding to a specific DNA motif) [8]. SNAI1 promotes epithelial–mesenchymal transition (EMT), which endows normal cells with cancer cell characteristics by repressing E-cadherin [9]. The targets of SNAI1 have been shown to be also involved in tumor development [10], immune repression [11] and tumor recurrence [12]. These studies suggest that SNAI1 is an oncogene that sustains onco-signalling pathways to boost tumor progression.

MicroRNAs (miRNAs) are approximately 20 to 22 nucleotides of non-coding RNA that cleave messenger RNA(mRNA) or repress mRNA translation in plants and animals [13]. Several studies have shown that altered miRNA expression is involved in glioma initiation, progression and recurrence via regulating cell apoptosis, proliferation, tumorigenesis, invasion, and metastasis [14]–[18]. Previously, we demonstrated that miR-128 is decreased in gliomas and inhibits cell proliferation, tumor growth and angiogenesis [19]. Most studies on miRNA tend to work within the typical model of ‘microRNA–mRNA’ interaction. However, information on the ‘transcription factor–microRNA–mRNA’ model in gliomas is currently lacking.

In our study, the mechanism of how the SNAI1/miR-128/SP1 axis facilitates glioma progression was investigated. Downregulation of SNAI1 increased miR-128 expression level through the reduction of transcriptional repression and a consequent decrease in SP1 protein level. Cell cycle, invasion and proliferation were restrained by the application of SNAI1 shRNA in glioma cell lines. Downregulation of SNAI1 inhibited SP1 protein level to a greater degree than the application of SNAI1 shRNA and miR-128 anti-sense oligonucleotide (ASO). Cell cycle retardation, proliferation and invasion suppression by SNAI1 shRNA were all alleviated by miR-128 ASO transfection, compared with the SNAI1 shRNA group. We propose that this signal axis may be a potential target for malignant glioma treatment.

## Results

### SNAI1 expression is correlated with glioma WHO grade

**Figure 1:**
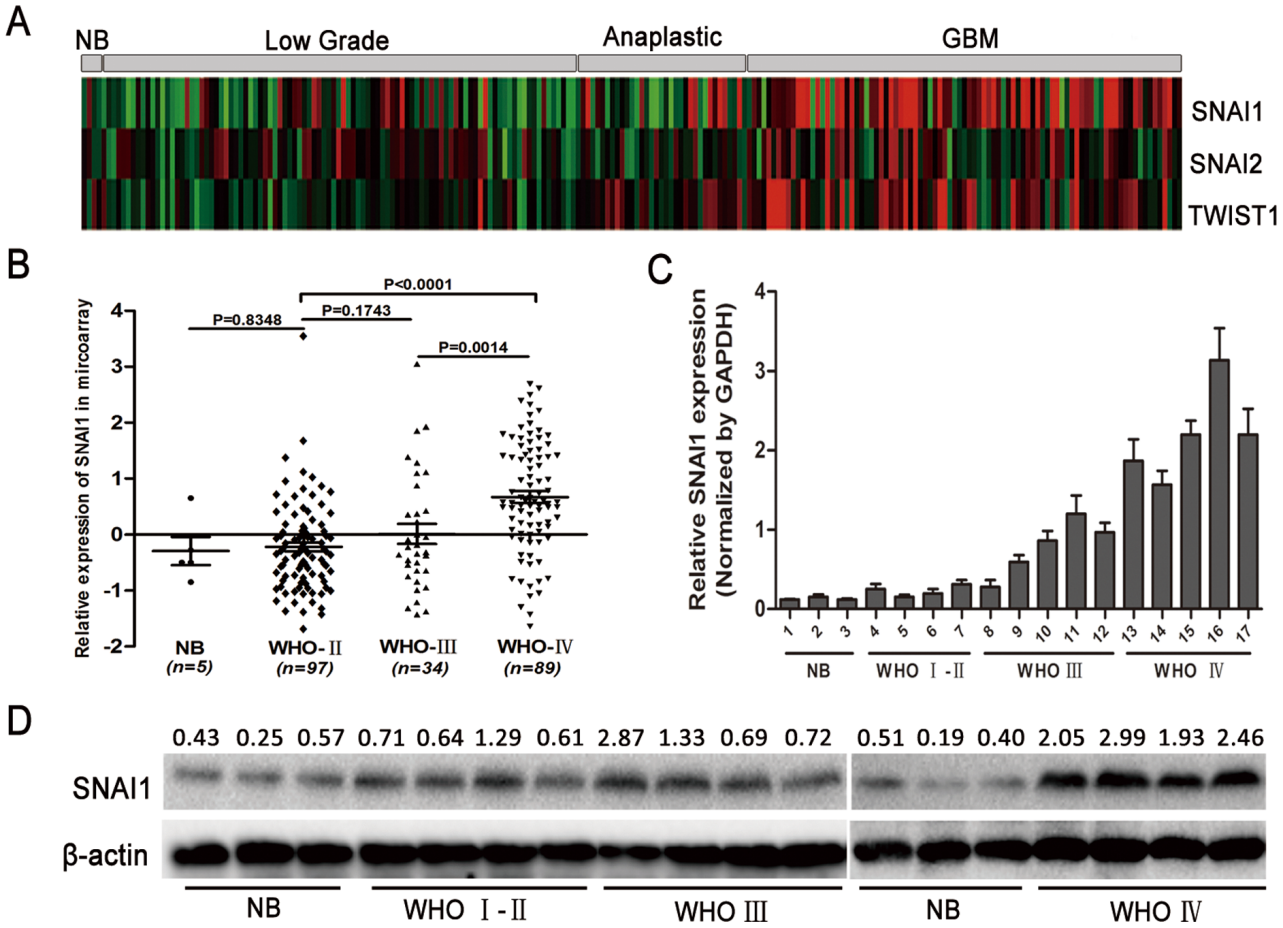
Expression profiles of SNAI1 in glioma. (A) A heat map delineating the expression of SNAI1 and epithelial–mesenchymal transition (EMT) correlated genes in 5 normal brain tissues and 220 glioma tissues (97 cases of WHO grade II, 34 cases of WHO grade III and 89 cases of WHO grade IV). (B) SNAI1 is correlated with glioma WHO grade, as shown in the scatter diagram. (C) and (D) mRNA and protein levels of SNAI1 in normal and glioma samples, the western blot band was quantified using Image Lab 4.0 software (Bio-Rad) and shown as the ratio of SNAI1/β-actin band intensity.

After investigating the Chinese Glioma Genome Atlas (CGGA) data, we found that SNAI1 expression increases in glioma tissues (WHO grade II to IV) in comparison with normal brain (Figure 1A). In particular, SNAI1 expression is significantly elevated in glioblastoma (Figure 1B). The mRNA and protein expression levels of SNAI1 were detected using normal brain tissues and glioma (WHO grade II to IV) tissues (Figure 1C and 1D) and the results were consistent with the CGGA data.

### SNAI1 function in glioma cell lines

**Figure 2:**
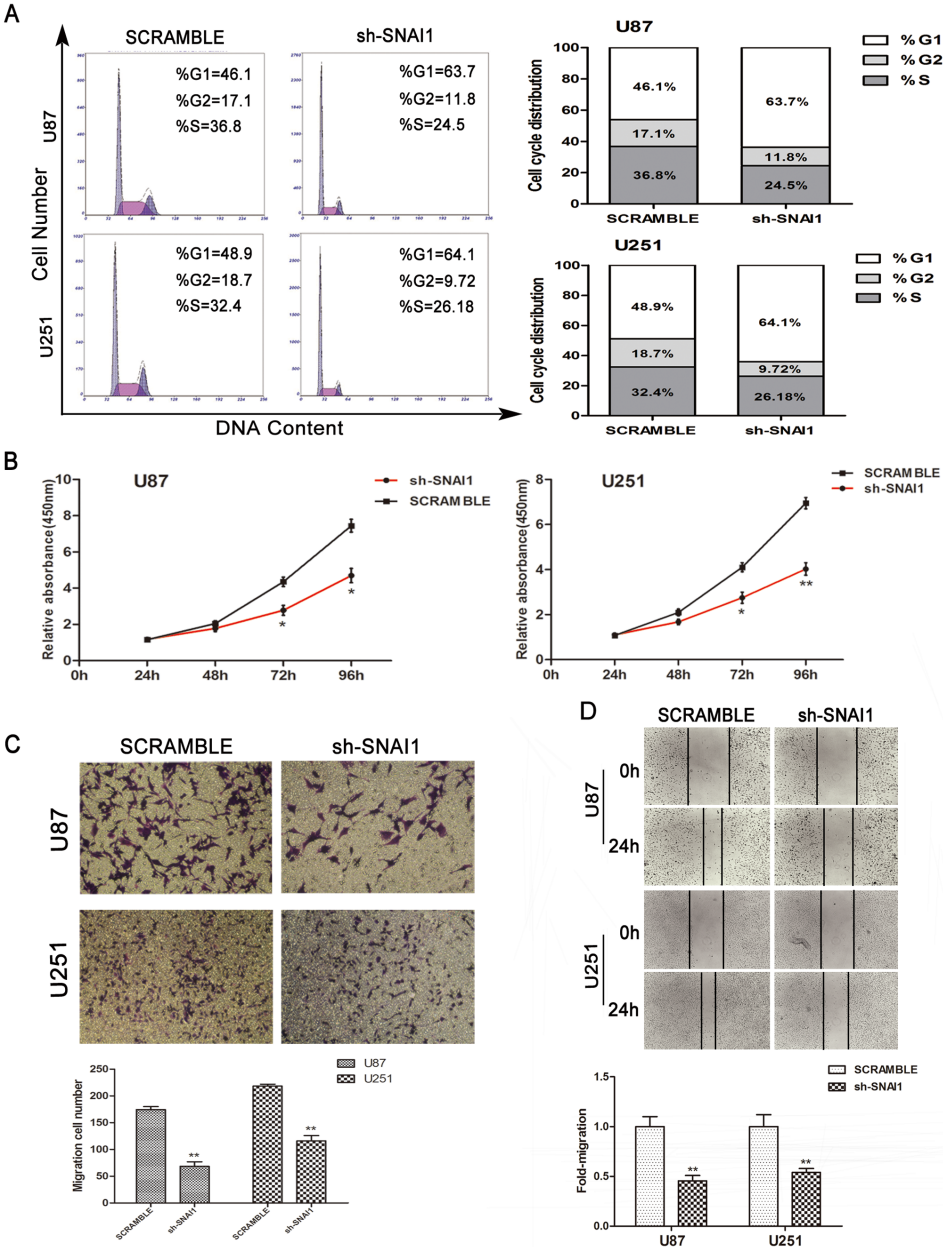
Role of SNAI1 on glioma U87 and U251 cell lines tumor biology. Results of in vitro assays are presented. SNAI1 knockdown suppresses cell cycle, as shown by flow cytometry (A); proliferation, as shown by CCK-8 (B); invasion, as shown by transwell invasion assay (C); and migration, as shown by wound-healing assay (D). Data are expressed as the mean ± S.D.*P<0.05, **P<0.01.

Most studies of SNAI1 focus on EMT. Informations about the effects of SNAI1 on the cell cycle, proliferation and invasiveness are lacking. In this study, we focused on the function of SNAI1 in the regulation of cell cycle, proliferation and invasiveness. Knockdown of SNAI1 by shRNA was validated by western blot (Figure S1). Flow cytometry showed that downregulation of SNAI1 resulted in G0/G1 phase retardation (Figure 2A). Cell counting kit-8 assay also revealed significant proliferation restraint of glioma cells (Figure 2B). Cell invasion in vitro was reduced by a wide margin (Figure 2C) and wound-healing assay showed decreased migration capacity (Figure 2D).

### miR-128b is a direct target of SNAI1

**Figure 3:**
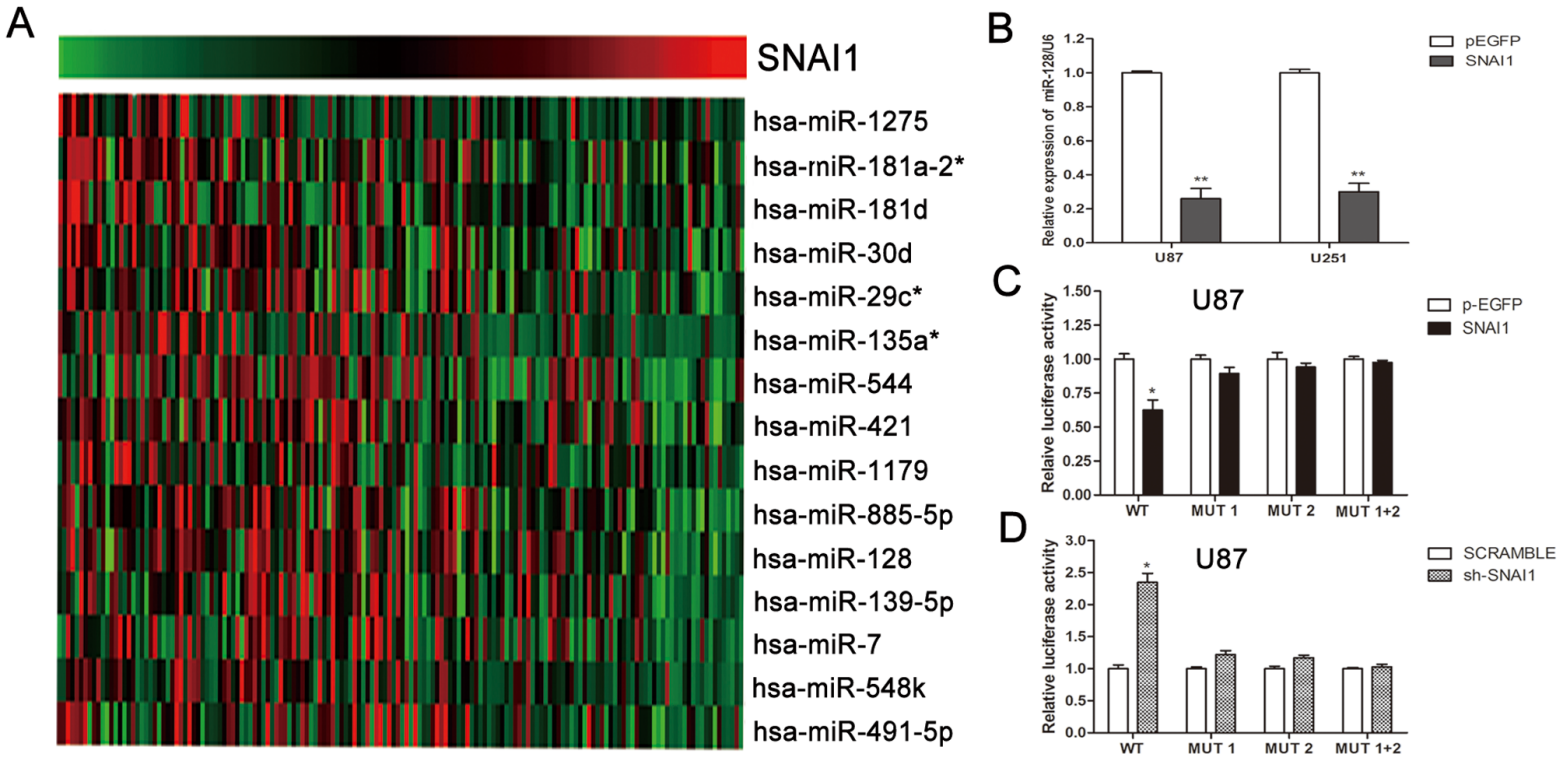
SNAI1 regulates miR-128b expression at the transcriptional level. (A) The heat map of 158 glioma sample microarray data reveals a negative correlation between SNAI1 and 15 miRNAs, including miR-128. (B) SNAI1 plasmid reduces miR-128 expression compared to the negative control group. (C) and (D) Luciferase reporter assays were used to identify the transcription-inhibition effect of SNAI1 on the promoter region of miR-128b. U87 cells were transfected with SNAI1 plasmid or SNAI1 shRNA along with different promoter constructs (wild or mutant type), and renilla luciferase was used as an internal control. After 48 h, luciferase assays were performed. Data are expressed as the mean ± S.D.*P<0.05, **P<0.01.

We analysed the CGGA microarray data of 158 glioma tissues using Matlab software to explore the potential relationship between SNAI1 and miRNA expression. We found that some miRNAs showed negative correlation with SNAI1 expression (Figure 3A and Table 1). Among the miRNAs identified, miR-128 was previously studied and proven to be a tumor suppressor in gliomas [19]. Therefore, the present study focused on miR-128.

**Table 1:** SNAI1 specific miRNA signature in human glioma tissues (negative correlation).

miRNA ID R value P value		
hsa-miR-885-5p	−0.3618	3.00E-06
hsa-miR-135a*	−0.3298	2.32E-05
hsa-miR-128	−0.32755	2.66E-05
hsa-miR-181a-2*	−0.32145	3.82E-05
hsa-miR-548k	−0.3051	9.70E-05
hsa-miR-30d	−0.3037	0.00010479
hsa-miR-1275	−0.27834	0.00039831
hsa-miR-544	−0.27428	0.00048776
hsa-miR-29c*	−0.27242	0.00053453
hsa-miR-1179	−0.26832	0.0006528
hsa-miR-181d	−0.26825	0.00065493
hsa-miR-139-5p	−0.26232	0.0008694
hsa-miR-421	−0.2603	0.0009565
hsa-miR-491-5p	−0.25885	0.0010235
hsa-miR-7	−0.25449	0.0012517

We tested the relationship between SNAI1 and miR-128 in glioma cell lines by transfecting SNAI1 shRNA and scramble oligonucleotides into U251 and U87 cells. The quantitative real-time PCR (qRT-PCR) results uncovered that miR-128 expression in the SNAI1 shRNA group increased when compared with that of the scramble group (Figure 3B). This result shows that SNAI1 can negatively regulate miR-128.

Previous studies have identified the SNAI1-binding site in the promoter of miR-128b [23]. Luciferase plasmids containing the wild type miR-128b promoter region and the corresponding mutant motifs were constructed. Luciferase assay results showed that in the SNAI1 shRNA transfected group, luciferase activity increased when compared with the scramble group, but only the increase in the wild type group was statistically significant. The SNAI1 plasmid and p-EGFP empty plasmid were also applied for luciferase assay. The results from U87 cell are shown in figure 3C and 3D and the U251 luciferase assay data is included in the Figure S2.

### MiR-128 targets SP1 by binding to its 3′-untranslated region (3′-UTR)

**Figure 4:**
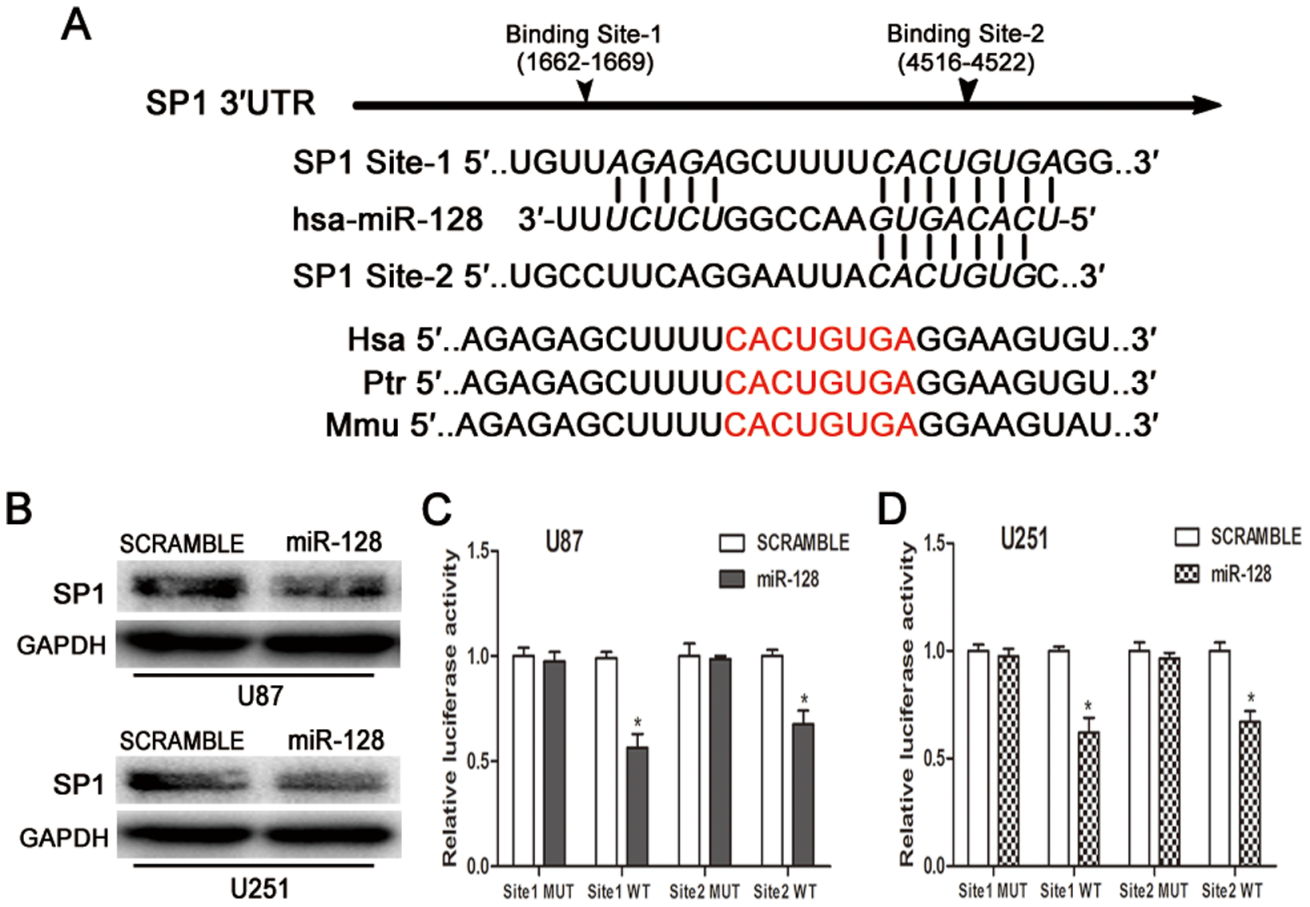
MiR-128 directly targets SP1. (A) Putative binding site of miR-128 within SP1 3′-UTR in human, chimpanzee and mouse, as predicted by TargetScan, PicTar, and MiRDB algorithms. (B) SP1 protein levels in U87 and U251 cells were detected after introduction of miR-128 mimics or scramble oligonucleotides. (C) and (D) In U87 and U251 cells, wild-type groups displayed decreased luciferase activity in comparison with mutant type groups. Data are expressed as the mean ± S.D. *P<0.05.

miRNA functions by targeting mRNA to inhibit translation. We investigated the direct targets of miR-128 using the TargetScan, PicTar and MiRDB algorithms. SP1 was selected for further analysis as there have been many studies on its role in cell cycle, tumor formation, growth and migration [24]–[26], but with very few reports in the context of gliomas. We initially increased miR-128 ectopic expression in glioma cell lines (Figure S3). We identified the binding sites of miR-128 within the 3′-UTR of SP1 mRNA and found that the binding motifs were conserved among species, such as human, chimpanzee and mouse (Figure 4A). Western blot analysis showed that ectopic expression of miR-128 reduced SP1 protein level (Figure 4B). Plasmids harboring WT or mutant 3′-UTR were synthesized. The results of luciferase assay demonstrated that luciferase activity in the co-transfected WT plasmid and miR-128 mimic groups decreased compared with the scramble group. The mutant plasmids groups showed no differences when compared with the scramble group (Figure 4C and 4D). The CGGA data showed that Pearson correlation between miR-128 and SP1 was negative (R = −0.3543, P<0.0001) (Figure S4).

### SNAI1/miR-128/SP1 axis in glioma tissues

**Figure 5:**
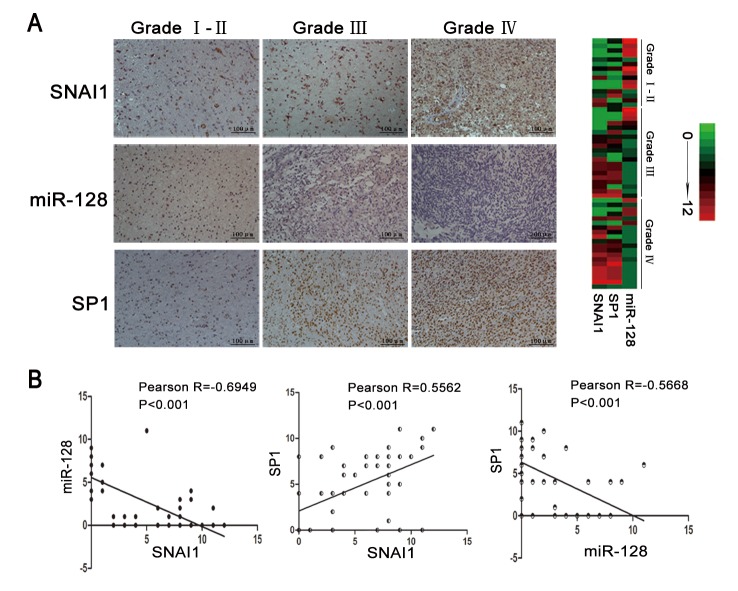
Expression of the SNAI1/miR-128/SP1 axis in glioma tissues. (A) SNAI1, miR-128 and SP1 were detected in 55 glioma samples by immunohistochemistry and in situ hybridisation staining. Some representative images are shown. Scale bar  = 100 µm. A heat map representing gene expression level was created with a scope of 0 to 12 to highlight differences within this range. (B) By quantification of immunohistochemistry and in situ hybridisation slides, we found that the Pearson correlations between SNAI1, SP1 and miR-128 were R_snai1 and miR-128_ = −0.6949 (p<0.001), R_snai1 and sp1_ = 0.5562 (p<0.001) and R_sp1 and miR-128_ = −0.5668 (p<0.001).

To assess expression level of the three elements of SNAI1/miR-128/SP1 axis in glioma tissues, 55 tissues (15 grade I to II, 20 grade III, 20 grade IV) were used for immunohistochemistry (IHC) and in situ hybridisation (ISH) staining. IHC showed that the SNAI1 and SP1 staining of grade I to II (low grade gliomas) was weaker than that of grade III and IV (high grade gliomas) tissues. ISH staining showed an opposite pattern, with low-grade gliomas showing stronger staining compared to high-grade gliomas (Table 2). Representative ICH and ISH images are presented in Figure 5A.

**Table 2:** SNAI1, SP1 and miR-128 expression in human glioma tissues.

Tissue	Number	The expression of SNAI1					The expression of SP1					The expression of miR-128				
		-	+	++	+++	Positive Rate(%)	-	+	++	+++	Positive Rate(%)	-	+	++	+++	Positive Rate(%)
I-II	15	5	6	3	1	66	7	5	2	1	53.3	3	4	6	2	80
III	20	5	3	9	3	75	5	4	9	2	75	9	8	2	1	55
IV	20	3	4	5	8	85	4	3	7	6	80	12	4	4	0	40

Negative expression (−), weak expression (+/1–4), moderate expression (++/5−8), strong expression (+++/9−12).

We then tested for possible correlations among SNAI1, miR-128 and SP1 expression levels. Pearson correlation of SNAI1 and miR-128 expression levels was negative (R = −0.6949, P<0.001). The same result was found for miR-128 and SP1 (R = −0.5668, P<0.001). Correlation between SNAI1 and SP1 showed a positive index (R = 0.5562, P<0.001) (Figure 5B).

### MiR-128 functions as extinguisher in the SNAI1/miR-128/SP1 axis

**Figure 6:**
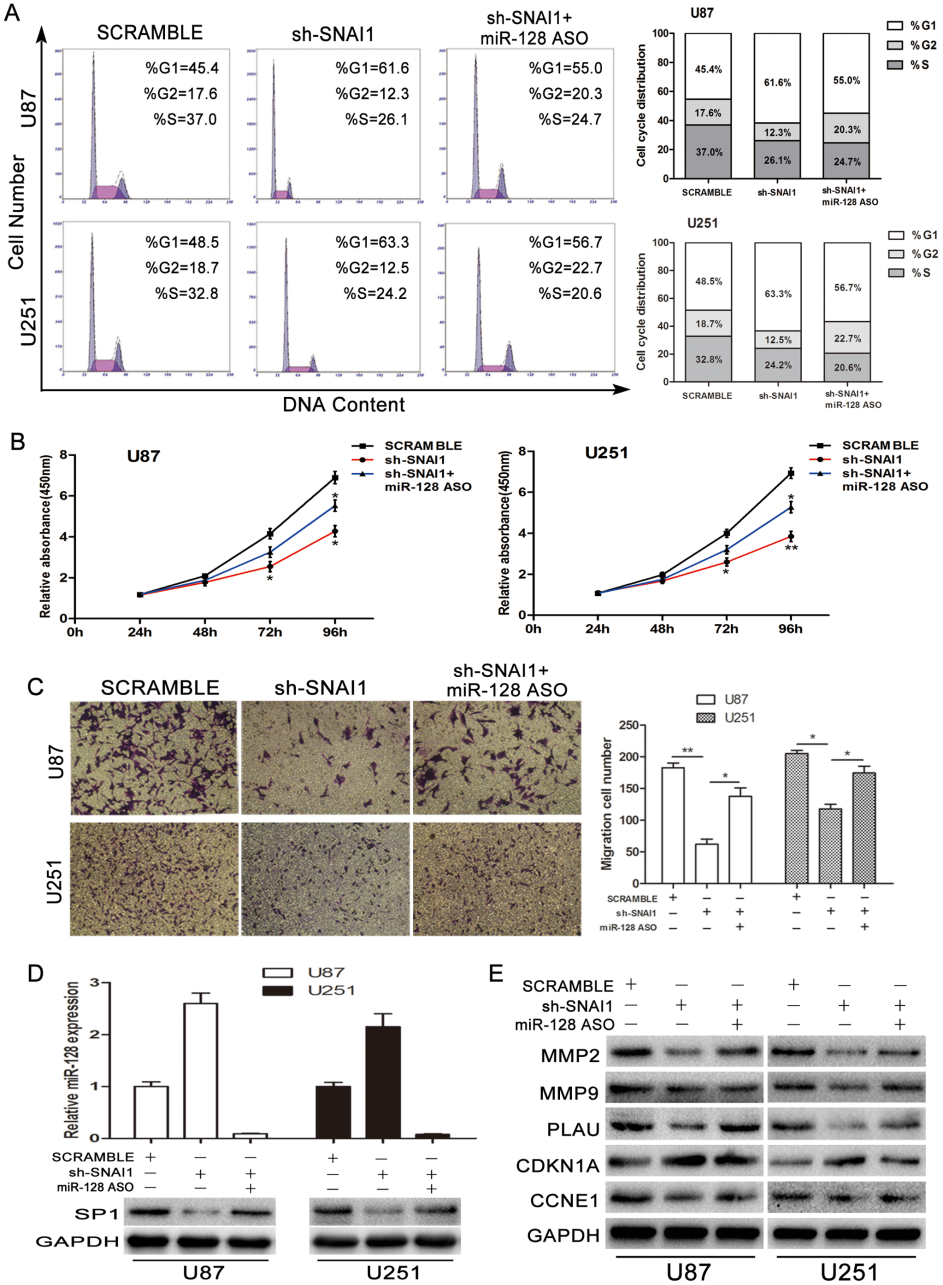
Effect of miR-128 on the SNAI1/miR-128/SP1 axis. U87 and U251 cells were differentially treated with SNAI1 shRNA and miR-128 ASO. (A), (B) and (C) miR-128 ASO weakened SNAI1 shRNA-induced cell cycle retardation, proliferation inhibition and invasion suppression. (D) miR-128 expression of cells treated with SNAI1 shRNA and SNAI1 shRNA plus miR-128 ASO were shown by quantitative real-time PCR. Decrease in SNAI1 shRNA-induced SP1 protein level was mitigated by the introduction of miR-128 ASO. (E) Protein level of several genes involved in cell cycle and proliferation, cell invasion and migration were analysed by western blot. Data are expressed as the mean ± S.D.*P<0.05, **P<0.01.

Many studies reported that miR-128 is reduced in several kinds of cancer [19], [23], [27], [28]. However, its functions in gliomas need further study. Primarily, miR-128 ASO antagonised SNAI1 shRNA function in glioma cell lines. U87 and U251 were transfected with scramble oligonucleotides (group A), SNAI1 shRNA (group B) and SNAI1 shRNA plus miR-128 ASO (group C). Cell cycle assay revealed that miR-128 ASO reduced the impact of sh-SNAI1 on cell cycle, specifically the G0/G1 phase (Figure 6A). CCK-8 assay showed that the proliferation rate of group C was higher than that of group B but lower than that of group A (Figure 6B). In vitro invasion assay showed similar results that were consistent with the cell cycle results (Figure 6C). Next, the RNA level of miR-128 and SP1 protein level were analysed using qRT-PCR and western blot analysis within groups A, B and C. The results are shown in figure 6D. The Pearson correlation of SNAI1 and SP1 in CGGA data was R = 0.2128 (P = 0.0015) (Figure S5). Furthermore, we investigated several genes including MMP2, MMP9, CDKN1a, CCNE1 and PLAU, which were related to cell cycle and proliferation, cell invasion and migration using western blot analysis (Figure 6E). Pearson correlation testing of the CGGA data revealed the following correlations, SNAI1 and MMP2 (R = 0.2758, P<0.0001), SNAI1 and MMP9 (R = 0.5785, P<0.0001), SNAI1 and CCNE1 (R = 0.2771, P<0.0001), SNAI1 and PLAU (R = 0.6910, P<0.0001) (Figure S6).

## Discussion

SNAI1 comes from the zinc-finger superfamily of SNAIL, which is dysregulated in numerous neoplasms [29]–[31]. SNAI1 participates in EMT and is the most essential transcription repressor of E-cadherin [32]. The CGGA data showed that SNAI1 was up-regulated in glioma tissues, especially in WHO grade IV gliomas in comparison with normal brain tissues. This result was confirmed by detecting the mRNA and protein expression levels in normal and glioma tissues. Apart from EMT, cell biological processes, such as cell cycle, proliferation, invasiveness and metastasis [33]–[35] are also affected by SNAI1. In our study, we found that reduced SNAI1 expression by shRNA suppressed the cell cycle, proliferation, invasiveness and migration of glioma cell lines. Thus, SNAI1 is critical for glioma tumor biology.

As a repressive transcription factor, SNAI1 generally represses genes coding target proteins via binding to specific motif. Aside from targeting the protein coding gene, SNAI1 may regulate other factors such as miRNA. Similar to protein coding genes, miRNA is subject to regulation, including transcriptional regulation, genomic amplification, processing, editing and decay [36]. Transcriptional regulation is believed to be a major factor in controlling miRNA expression. Mestdagh et al found that MYCN/c-MYC binds to the promoters of the miR-17-92 cluster, miR-214, miR-15b and miR-130a [37]. Pietro Laneve et al showed that REST and CREB control miR-9-2 expression by binding to a conserved region in its promoter during human neuronal differentiation [38]. In this study, we demonstrate that miR-128b is directly suppressed by SNAI1 by binding to the promoter region of miR-128b in gliomas.

MiR-128 was initially shown to be enriched in brain and frequently decreased in glioma cells [39], and subsequently in neuroblastoma, prostate, breast cancer, and demonstrated to be a tumor suppressor gene [23], [27], [28]. Deregulation of miR-128 leads to malignant characteristics, including enhanced proliferation [40], apoptosis resistance [41], invasion and metastasis activation [27] and promotion of self-renewal[15]. In the current study, we demonstrated that miR-128b is a direct target of SNAI1 and showed that miR-128 can override the effect of SNAI1 on glioma cell cycle, proliferation and invasiveness. Subsequently, we found that SP1 expression is restrained by miR-128 via 3′-UTR and that SNAI1 regulates SP1 partly via miR-128. SP1 has a wide variety of functions that favor generation of neoplasms [42]. In addition to SP1, tumor supporters such as E2F3a, BAX, BMI-1, DCX and Reelin are also targeted by miR-128 [15], [27], [40], [41]. These suggest that SNAI1 may affect the expression of many more oncogenes. In the SNAI1/miR-128/SP1 axis, miR-128 acts as a negative regulator, which resist the SNAI1-promoted progression of gliomas.

## Conclusion

**Figure 7:**
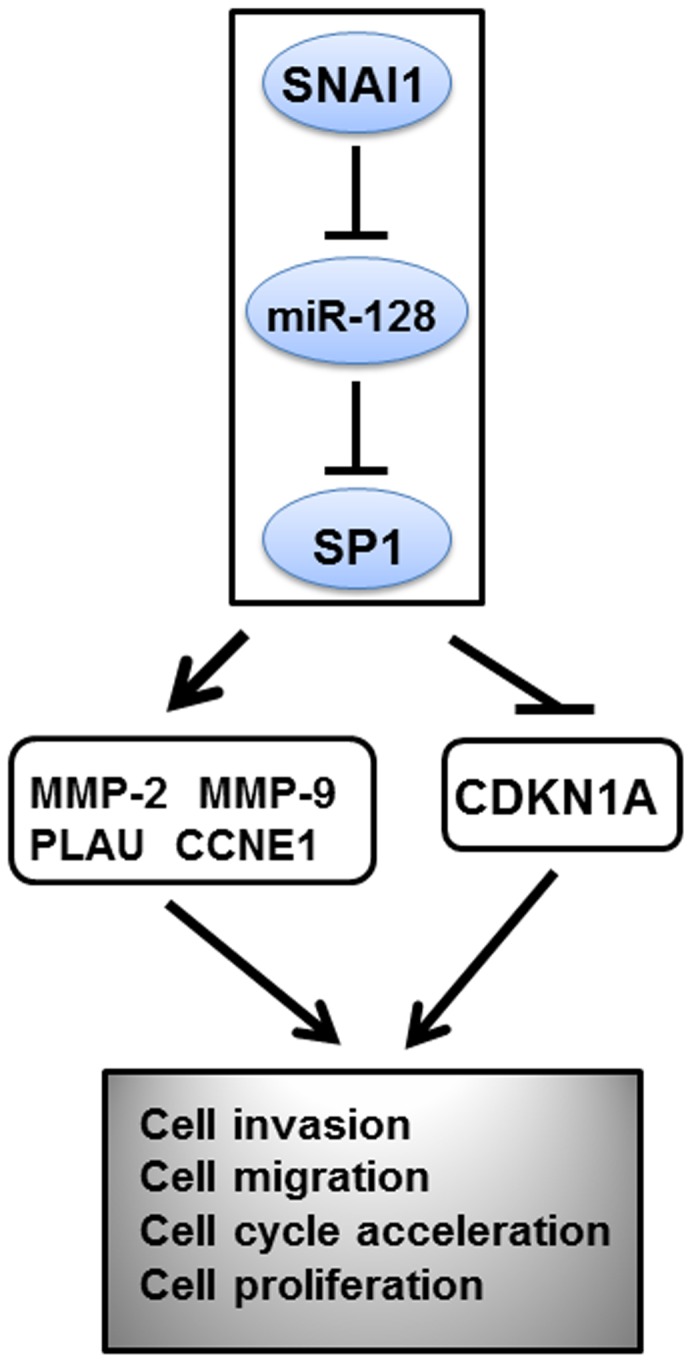
SNAI1/miR-128/SP1 axis regulates glioma progression. SNAI1 suppresses miR-128 expression and miR-128 restrains SP1 expression. SNAI1 increases SP1 expression through miR-128. The SNAI1/miR-128/SP1 axis promotes cell invasion, cell migration, cell proliferation and cell cycle via increasing MMP-2, MMP-9, PLAU and CCNE1 expression and reducing CDKN1A expression.

In general, our data revealed that upregulated SNAI1 accelerates glioma progression and suppresses the expression of miR-128, which can oppose SNAI1's effect and modulate SP1 expression. Our findings first establish a role for the SNAI1/miR-128/SP1 axis in the regulation of glioma evolution (Figure 7). Thus, we have expanded the current knowledge by delineating the function of the SNAI1 axis in glioma progression, and propose that this axis is a potential candidate molecular target for clinical prognosis or therapy.

## Materials and Methods

### Microarray analysis

Human tissue samples data are downloaded from Chinese Glioma Genome Atlas (CGGA) data portal (http://www.cgga.org.cn/portal.php). Microarray assay was performed as described previously [20].

### Human tissues

All human tissues were collected in The First Affiliated Hospital of Nanjing Medical University. All specimens were histopathologically diagnosed on the basis of World Health Organisation (WHO) classification. Specimens used for qRT-PCR and western blot analyses were stored in liquid nitrogen. 55 glioma tissues (15 grade I to II, 20 grade III and 20 grade IV) were used for immunohistochemistry and in situ hybridisation staining. This study was approved by the institutional review board of Nanjing Medical University, and written informed consent was obtained from all patients.

### Cell culture

The human glioma cell lines U251 and U87 (the Chinese Academy of Sciences, Shanghai, China) were cultured in Dulbecco's modified Eagle's medium(DMEM, Gibco) supplemented with 10% fetal calf serum (FBS, Gibco). The cells were maintained at 37°C in 5% CO_2_ condition.

### Oligonucleotides, plasmids and cell transfection

The oligonucleotides (GenePharma, Shanghai, China) were as follows: miR-128 mimics 5′-UCACAGUGAACCGGUCUCUUU-3′ (sense), 5′-AGAGACCGGUUCACGGUGAUU-3′ (anti-sense); scrambled miRNA (NC) 5′-UUCUCCGAACGUGUCACGUTT-3′ (sense), 5′-ACGUGACACGUUCGGAGAATT-3′ (anti-sense); miR-128 ASO 5′-AAAGAGACCGGUUCACUGUGA-3′ and miR-128 ASO (NC) 5′-CAGUACUUUUGUGUAGUACAA-3′. The SNAI1 shRNA and control shRNA plasmids were obtained from GenePharma (Shanghai, China). SNAI1 plasmid was purchased from ADDGENE (catalogue # 16225), and p-EGFP C2 empty plasmid was from Invitrogen. According to the product specification, the oligonucleotides were transfected into cells with Lipofectamine 2000 reagent (Invitrogen) and FuGENE 6 Transfection Reagent (Promega) were used for plasmid transfection.

### In vitro cell invasion and migration assays

Transwell assays were used to test cell invasion. Matrigel invasion chamber (Millipore Corporation) and Matrigel (BD Biosciences) were used. Transfected U87 and U251 cells were trypsinised, washed, re-suspended in serum-free DMEM and then seeded into the upper chamber membrane. Below the membrane, the chemo-attractant which is mixture of 500 µL DMEM medium and 20% FBS was added. After incubation for 24 h, cotton swabs were used to remove the unpenetrated cells, whereas the penetrated cells were stained with 0.1% crystal violet. Images were captured under inverted microscope (100×) (Nikon) for each well.

For wound-healing assays, after 24 h following transfection, when the cells had reached 90% confluence, a 200 µL pipette tip was used to scratch the monolayer cells. Cells were maintained with 4% (v/v) FBS-supplemented DMEM for the entire experiment. Images were captured (0, 16 and 30 h) under inverted microscope (40×) (Leica) to assess the cell migration ability.

### Cell counting kit-8 assay (CCK-8)

CCK-8 assay was applied for cell viability evaluation. Transfected cells were added to 96 well plates (3×10^3^ cells/well) with 100 µL medium. Wells with the medium alone (no cells) served as blank controls. CCK-8 solution (Shanghai Beyotime Biotechnology) (10 µL/well) was added and then incubated for 2 h. The 450 nm optical density was recorded using a microplate reader (Multiscan FC, Thermo Scientific) at different times (24, 48, 72 and 96 h) after transfection. Experiments were performed in triplicate.

### Cell cycle assay

Cells were first washed with phosphate-buffered saline (PBS). Next, 75% ethanol was used to cover the cells at −20°C overnight. Then the cells were washed with PBS and added in Hank's balanced salt solution including 50 µg/mL propidium iodide and 50 µg/mL RNaseA. Finally, the cells were incubated for 1 h at room temperature. Gallios flow cytometer (Beckman Countler) was used to analyse the samples. The data were analysed with Multi Cycle AV for Windows software and presented as percentages in different phases.

### Quantitative real-time PCR

Total RNA was extracted from human glioma specimens or cells by TRIzol reagent (Invitrogen) following the product specification. TaqMan real-time reverse transcription PCR were performed with ABI StepOnePlus system (Applied Biosystems, USA), and U6 was used for normalization. The miR-128 probe was purchased from Applied Biosystems. The primers used were TCGGAAGCCTAACTACAGCGA (forward) and AGATGAGCATTGGCAGCGAG (reverse) for SNAI1; GATTAGGCTTCCCCTCCCAT (forward) and TGGCCACTGTGCTCCTTTTAT (reverse) for SP1. The 2^–ΔΔCt^ method was applied for gene expression analysis.

### Western blot analysis

Western blot was carried out as previously described [17]. Primary antibodies were as follows: SNAI1 (1∶200, RnD systems), SP1 (1∶1000, Cell Signalling), CDKN1a (1∶1000, Cell Signalling), CCNE1 (1∶1000, Cell Signalling), MMP2 (1∶1000, Cell Signalling), MMP9 (1∶1000, Cell Signalling), PLAU (1∶150, Abcam), GAPDH (1∶5000, Bioworld) and β-actin (1∶5000, Bioworld).

### Luciferase reporter assay

The SP1 3′-UTR has two miR-128 binding sites: 1662 to 1669(AGAGA------CACUGUGA) and 4516 to 4522(CACUGUG). Two wild–type (WT) luciferase reporter plasmids and two mutants (ACACA------GAGUCUCA and GAGUCUC) were constructed and inserted into pGL3–control vector (Invitrogen). The miR-128b promoter region containing SNAI1-binding sequence (---CACATG---) was inserted into pGL3-basic vector along with the three corresponding mutants (---GTGTAC---) (Invitrogen), as previously described [23]. After 48 h following transfection, dual-Luciferase Reporter Assay System (Promega) was used to detect luciferase activity in accordance with product specification.

### Immunohistochemistry (IHC) and in situ hybridisation (ISH) staining

Paraffin-embedded glioma tissue sections were used to evaluate the expression levels of SNAI1 and SP1. 3% hydrogen peroxide was used to block endogenous peroxidase activity. The slides were incubated with primary antibodies (SP1 1∶2000 Cell Signalling and SNAI1 1∶40 RnD systems) overnight at 4°C, followed by secondary antibody (1∶200 Gene Tech, China) for 1 h at room temperature. The positive reaction was obtained using diaminobenzidine solution, followed by counter staining with hematoxylin. As for the negative control, PBS was used instead of the primary antibody [21]. Images were captured under inverted microscope (200×, Nikon NIS-Elements). In situ hybridisation was performed as described previously[22].

Evaluation of IHC and ISH staining was independently performed by two experienced pathologists blinded to the procedure. The percentage of positive tumor cells was scored as follows: 0 (<5% positive cells), 1 (6% to 25% positive cells), 2 (26% to 50% positive cells), 3 (51% to 75% positive cells) and 4 (>76% positive cells). The intensity of staining was scored semiquantitatively as follows: 0 (negative), 1 (weakly positive), 2 (moderately positive) and 3 (strongly positive). Multiplication of the intensity and percentage scores generated the aggregate staining score: 0 (negative), + (1–4), ++ (5–8) and +++ (9–12) [21].

### Statistical analysis

Statistical evaluation of the data was performed by *t*-test using SPSS 13.0 statistical software. Statistical significance was considered at P<0.05. Matlab software was used for Pearson correlation analysis. All experiments were performed three times.

## Supporting Information

Figure S1 **Transfection efficiency of sh-SNAI1 in U87 and U251 by western blot.** Each experiment was performed three times.
(TIF)

Figure S2 **Luciferase reporter assays were used to identify the transcription-inhibition effect of SNAI1 on The promoter region of miR-128b.** U251 cells were transfected with SNAI1 plasmid or SNAI1 shRNA along with different promoter constructs (wild or mutant type), and renilla luciferase was used as an internal control. After 48 h, luciferase assays were performed. Data were expressed as the mean ± S.D. *P<0.05. Each experiment was performed three times.
(TIF)

Figure S3 **Transfection efficiency of miR-128 anti-sense oligonucleotide in U87 and U251 by qRT-PCR.** Data were expressed as the mean ± S.D. **P<0.01. Each experiment was performed three times.
(TIF)

Figure S4 **Pearson correlation (R = −0.3543, P<0.0001) between miR-128 and SP1 in 158 glioma tissues of the CGGA data. The 158 glioma tissues data used was randomly chosen from the 220 glioma cases.**
(TIF)

Figure S5 **Pearson correlation (R = 0.2128, P = 0.0015) between SNAI1 and SP1 in 220 glioma tissues of the CGGA data.**
(TIF)

Figure S6 **Pearson correlation analysis of 220 glioma tissues of the CGGA data: SNAI1 and MMP2 (R = 0.2758, P<0.0001), SNAI1 and MMP9 (R = 0.5785, P<0.0001), SNAI1 and CCNE1 (R = 0.2771, P<0.0001), SNAI1 and PLAU (R = 0.6910, P<0.0001).**
(TIF)
