# Supplementary figures and images for: An Axis Involving SNAI1, microRNA-128 and SP1 Modulates Glioma Progression
Source: PLoS One. 2014 Jun 24;9(6):e98651. doi: 10.1371/journal.pone.0098651 (PMC4068992; doi:10.1371/journal.pone.0098651)

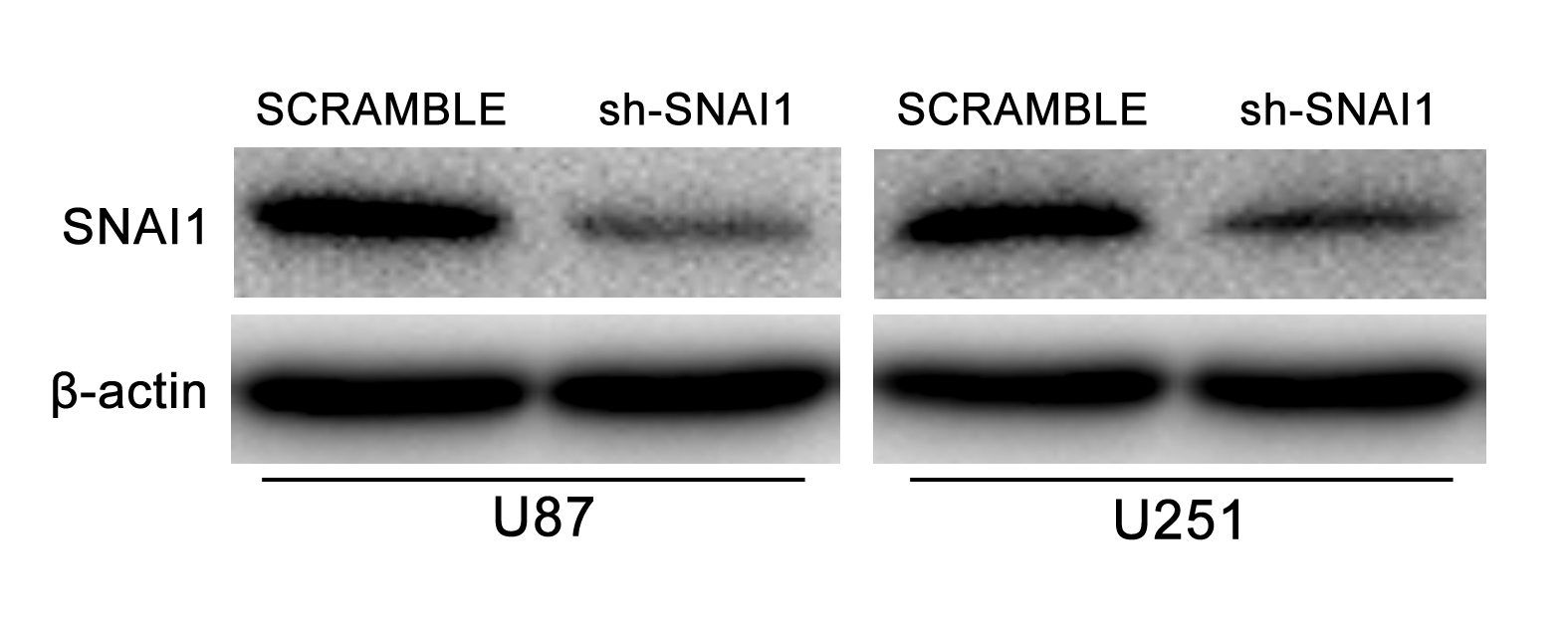

Supplement: Figure S1 — Transfection efficiency of sh-SNAI1 in U87 and U251 by western blot. Each experiment was performed three times. (TIF) [file pone.0098651.s001.tif]

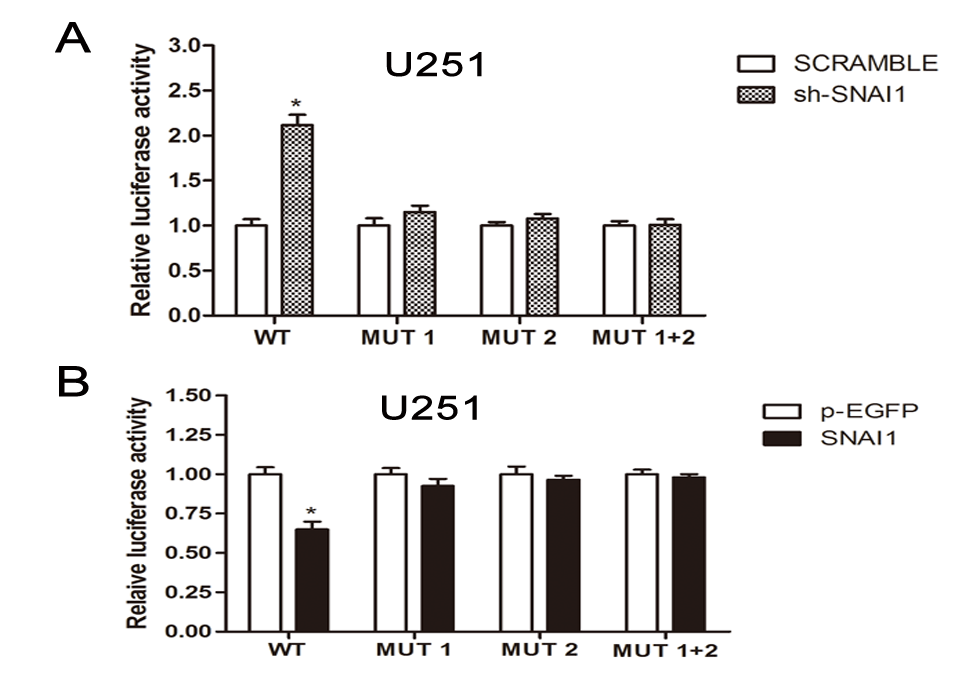

Supplement: Figure S2 — Luciferase reporter assays were used to identify the transcription-inhibition effect of SNAI1 on The promoter region of miR-128b. U251 cells were transfected with SNAI1 plasmid or SNAI1 shRNA along with different promoter constructs (wild or mutant type), and renilla luciferase was used as an internal control. After 48 h, luciferase assays were performed. Data were expressed as the mean ± S.D. *P<0.05. Each experiment was performed three times. (TIF) [file pone.0098651.s002.tif]

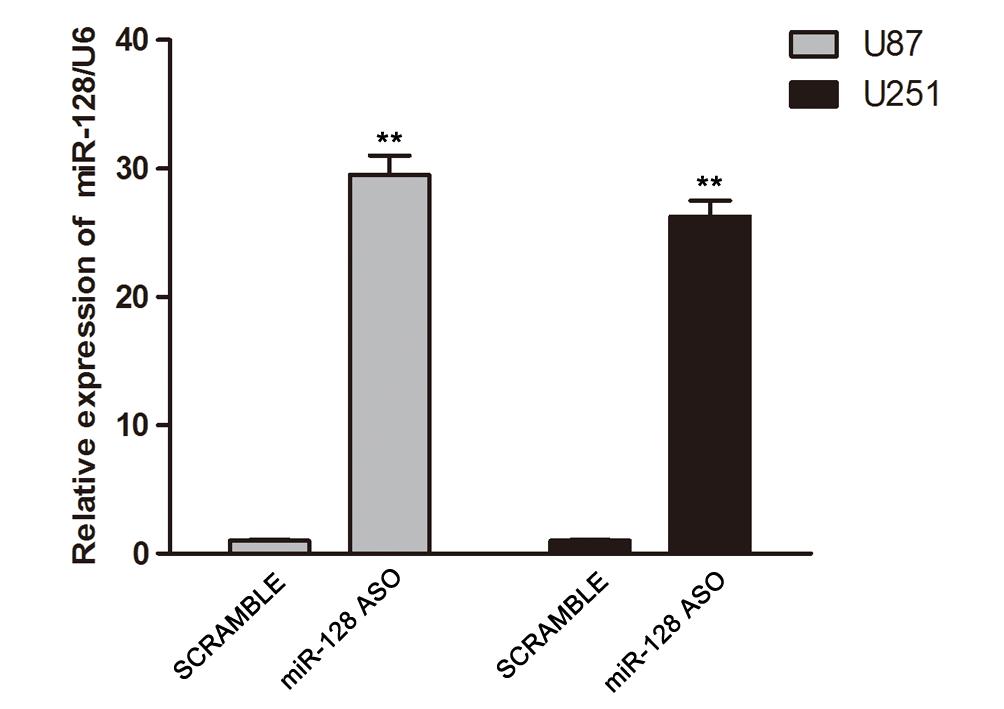

Supplement: Figure S3 — Transfection efficiency of miR-128 anti-sense oligonucleotide in U87 and U251 by qRT-PCR. Data were expressed as the mean ± S.D. **P<0.01. Each experiment was performed three times. (TIF) [file pone.0098651.s003.tif]

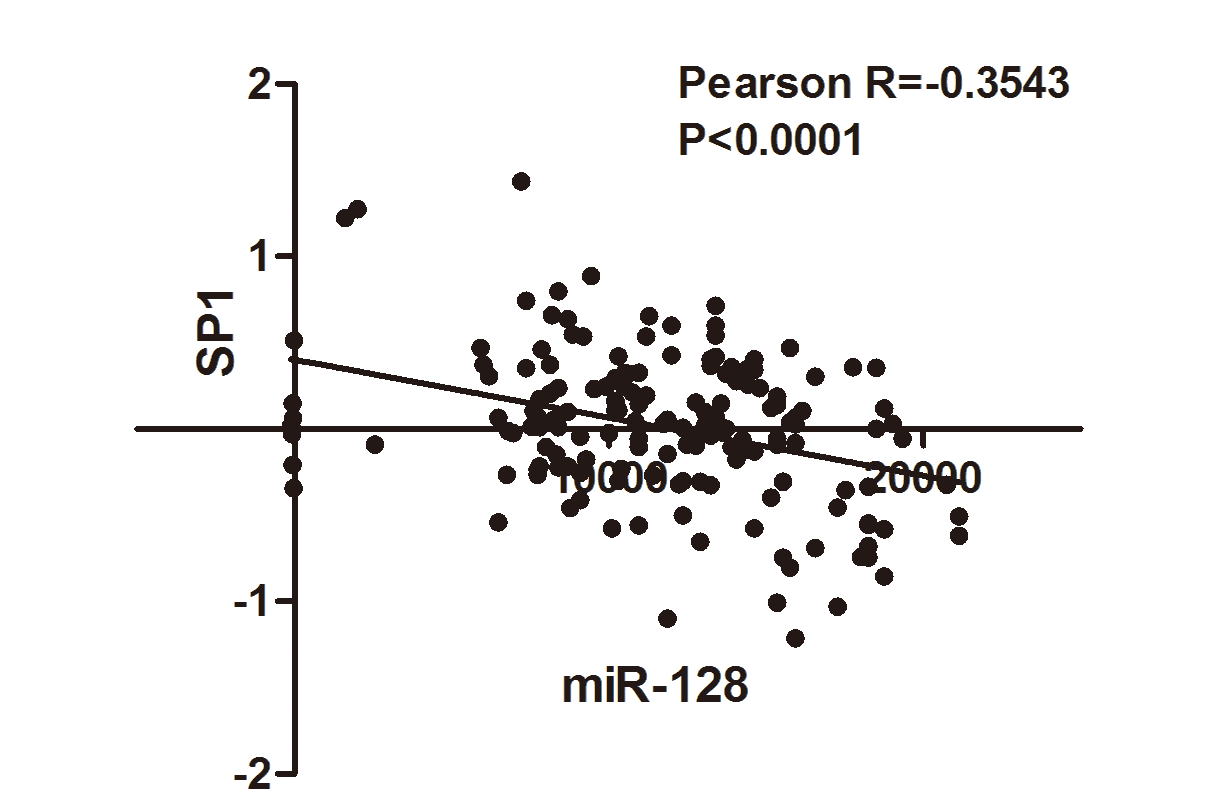

Supplement: Figure S4 — Pearson correlation (R = −0.3543, P<0.0001) between miR-128 and SP1 in 158 glioma tissues of the CGGA data. The 158 glioma tissues data used was randomly chosen from the 220 glioma cases. (TIF) [file pone.0098651.s004.tif]

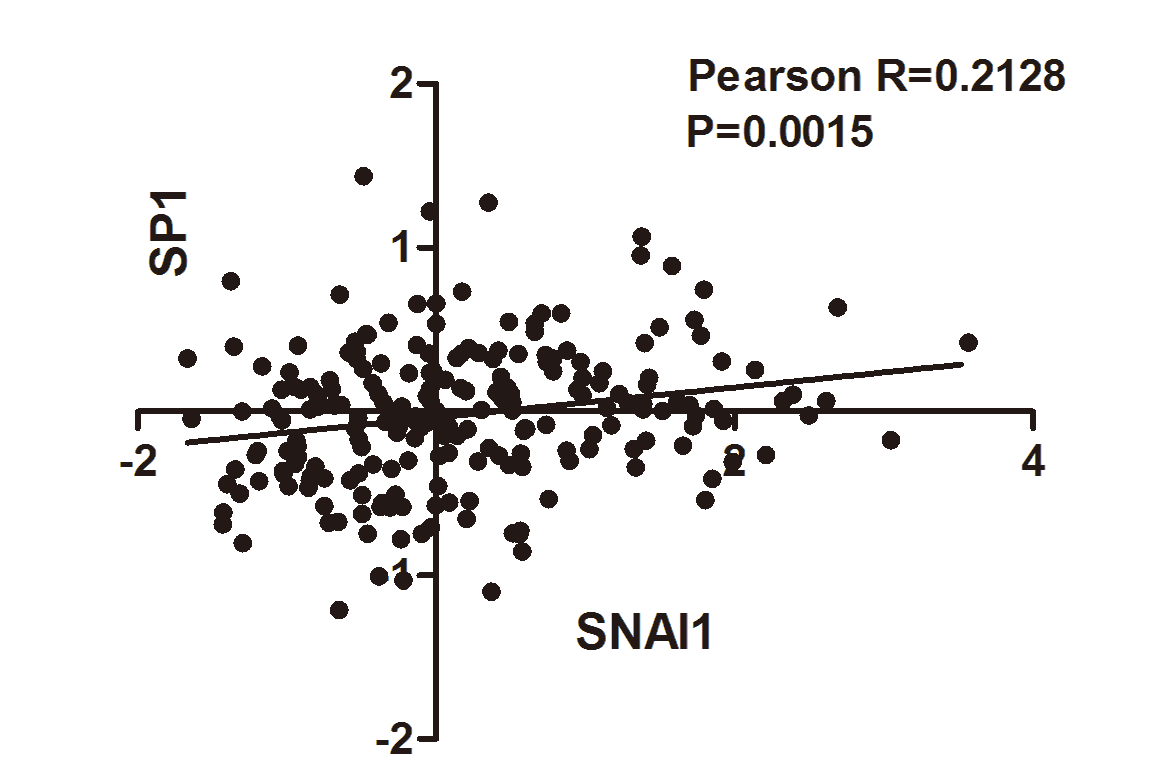

Supplement: Figure S5 — Pearson correlation (R = 0.2128, P = 0.0015) between SNAI1 and SP1 in 220 glioma tissues of the CGGA data. (TIF) [file pone.0098651.s005.tif]

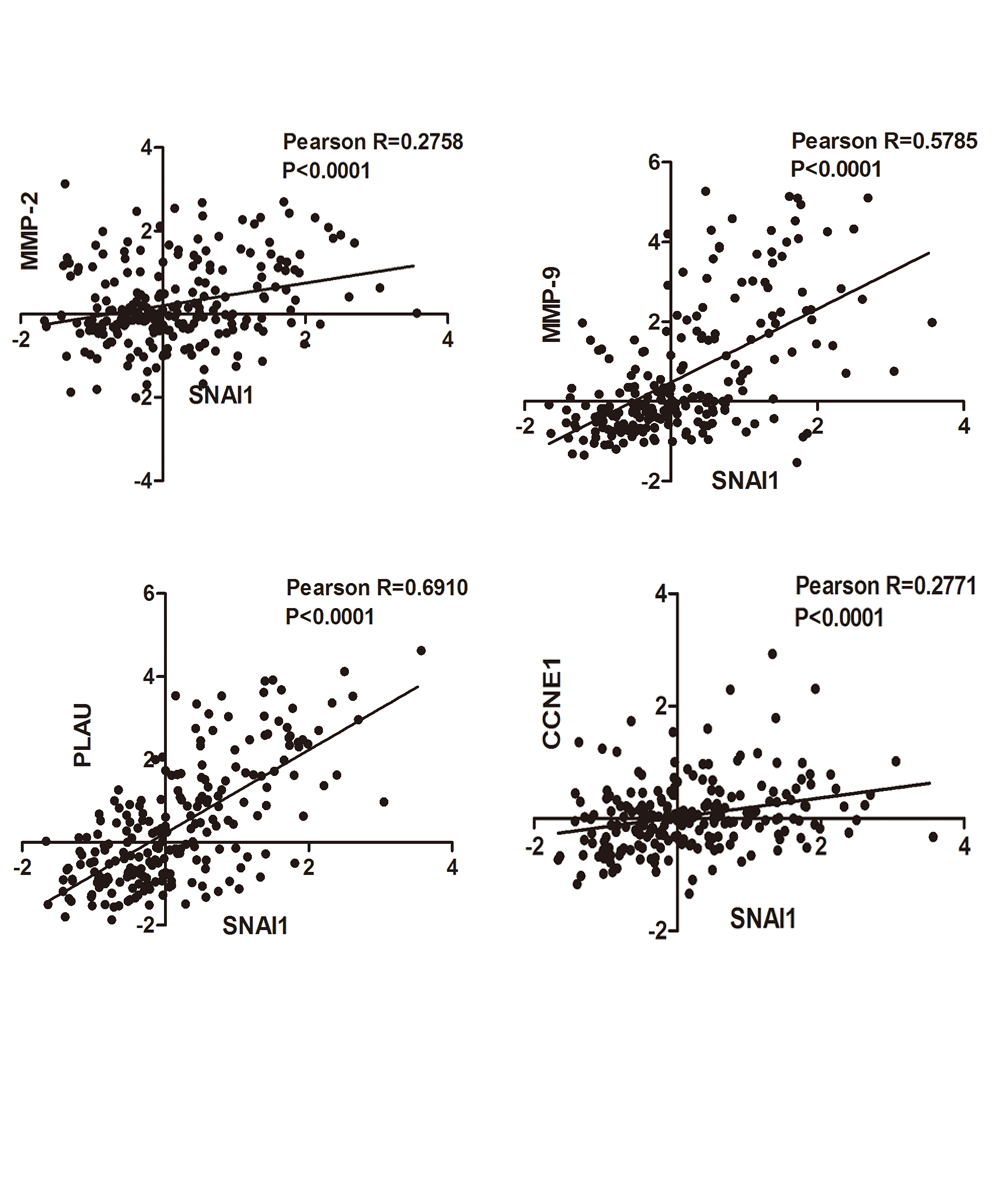

Supplement: Figure S6 — Pearson correlation analysis of 220 glioma tissues of the CGGA data: SNAI1 and MMP2 (R = 0.2758, P<0.0001), SNAI1 and MMP9 (R = 0.5785, P<0.0001), SNAI1 and CCNE1 (R = 0.2771, P<0.0001), SNAI1 and PLAU (R = 0.6910, P<0.0001). (TIF) [file pone.0098651.s006.tif]
